# Editorial: The impact of pharmacokinetics and pharmacodynamics on antimicrobial stewardship

**DOI:** 10.3389/fcimb.2025.1684355

**Published:** 2025-09-09

**Authors:** Zeynep Ture, Emine Alp Meşe, Joana Alves, Claire Roger, Rony M. Zeenny

**Affiliations:** ^1^ Private Gürlife Hospital, Infectious Diseases Clinic, Eskişehir, Türkiye; ^2^ Department of Infectious Diseases and Clinical Microbiology, Ankara Bilkent City Hospital Faculty of Medicine, Ankara Yıldırım Beyazıt University, Ankara, Türkiye; ^3^ Hospital de Braga, Braga, Portugal; ^4^ Anesthesiology and Critical Care Medicine Department, Nimes University Hospital, Place du Professeur Robert Debre, Nimes, France; ^5^ Department of Infectious Diseases, Hospital de Braga, Braga, Portugal; Local Unit of the Program for Prevention and Control of Infection and Antimicrobial Resistance, Hospital de Braga, Braga, Portugal

**Keywords:** antimicrobial stewardship, antimicrobial resistance, pharmacokinetics, pharmacodynamics, intensive care unit

Antimicrobial resistance (AMR) is one of the greatest threats to public health, and requires urgent, innovative strategies according to the World Health Organization. To overcome this global crisis, antimicrobial stewardship (AMS) programs have emerged as a key strategy aimed at preserving the effectiveness and extending the life of existing antibiotics by using the “right patient, right route of admission, right drug, right dose, right time.” It is becoming more widely acknowledged that conventional “one-size-fits-all” methods are inadequate, especially when dealing with multidrug-resistant (MDR) infections and critically ill patients who demonstrate considerable physiological variety (Gatti et al., 2025; Palmer et al., 2022). The integration of the principles of pharmacokinetics (PK) – what the body does to the drug – and pharmacodynamics (PD) – the effect of the drug on the pathogen – forms the scientific basis of modern AMS (Rodríguez-Gascón et al., 2021; Alikhani et al., 2025; Lanckohr et al., 2021). PK/PD integration aims to personalize treatment to maximize clinical effect while minimizing the risk of toxicity and resistance development (Gatti et al., 2025).

This Research Topic of Frontiers in Cellular and Infection Microbiology, “The Impact of Pharmacokinetics and Pharmacodynamics on Antimicrobial Stewardship,” brings together valuable studies, ranging from theory to practice, that illuminate the most current aspects of this dynamic and critical field. This Research Topic covers five key articles that reflect the multifaceted nature of PK/PD-based AMS, complementing each other and offering significant contributions to this field. These articles demonstrate why treatment should be individualized, how this can be achieved technologically, and how to overcome the complex nature of resistance.

## Why should treatments be individualized?

Optimal dose is achieved understanding PK/PD variations through special patients’ populations (elderly, pediatric, patients with obesity, critically ill or patients with immunosuppression), to specific host characteristics such as volume of distribution, renal clearance, weight, organ support, also minimum inhibitory concentration (MIC) of the pathogen, and the site and severity of the infection. Two studies demonstrated how misleading the concept of “standard dose” can be, particularly in sensitive populations and for drugs with narrow therapeutic ranges. The study presented by Chen et al., evaluated the serum concentrations of voriconazole in patients with hematological malignancies. They revealed that concentration varied significantly not only by route of administration but also by polymorphisms in genes such as the CYP2C19 enzyme that metabolizes the drug. This means that the same dose can lead to subtherapeutic or toxic levels in patients with different genetic polymorphisms. Hu et al. investigated the pharmacokinetics of polymyxin B in elderly patients. This study demonstrated that even standard doses above the recommended therapeutic range lead to potentially nephrotoxic exposures. Age-related physiological changes, particularly decreased renal function, slow the drug’s excretion, leading to its accumulation. This study also directly contributes to clinical practice by providing a reliable and practical high-performance liquid chromatography-tandem mass spectrometry (HPLC-MS/MS) method for such critical monitoring. These two studies demonstrate why inter-individual and inter-population variability makes therapeutic drug monitoring and model-based approaches indispensable for ensuring treatment success and safety.

## How can it be achieved technologically?

Once the need for individualized treatment is recognized, the question of how to implement it in clinical practice arises. Two articles in this Research Topic address this question with technological innovations. Zmerli et al. presents a “proof of concept” showing that, using benchtop scanning electron microscopy (SEM), the bactericidal or bacteriostatic effect of antibiotics can be visually estimated in as little as 1 to 2 hours. Given the 18–48 hour waiting period of traditional culture and susceptibility testing, this technology has the potential to take diagnostic stewardship to the next level, providing critical information about the effectiveness of empirical therapy within hours. The study by Yoon et al. demonstrates the potential for translating this rapid diagnostic information into action. The researchers developed a population PK/PD model for vancomycin, to achieve efficacy and prevent major adverse events. This model, using a Model-Informed Precision Dosing approach, allows for the determination of optimal dosing regimens and treatment durations based on individual patient characteristics (age, kidney function, etc.). The R Shiny-based web application they developed transforms this complex pharmacometrics information into a visual, understandable, and actionable format for clinicians, embodying the role of technology in clinical decision support. These two studies represent two key technological pillars of AMS: rapid diagnosis and smart dosing. The synergy of these technologies could enable clinicians to make the correct pathogen-drug match within hours and use this information to create a personalized, optimized treatment plan at the bedside, both improving clinical outcomes and reducing healthcare costs (Hall et al., 2024).

## How can the complex structure of AMR be explored?

Antimicrobial stewardship also requires understanding of the evolution of resistance and developing strategies to counter it. Li et al.‘s study highlights the complexity of this challenge and the critical role of PK/PD in this area. The study examined the development of heteroresistance in carbapenem-resistant *Klebsiella pneumoniae* isolates to a new and potent antibiotic, ceftazidime-avibactam. Heteroresistance is the development of a small subpopulation of bacteria within a bacterial population that becomes resistant to a drug and is often overlooked by standard susceptibility testing. The researchers found that this resistance stems from overexpression of the blaKPC gene, which encodes the beta-lactamase enzyme, rather than from *de novo* mutations. This is a dynamic process in which, under selective drug pressure, the resistant subpopulation is selected and proliferated, leading to treatment failure.

The common message across articles in this Research Topic is that the future of AMS consists of a personalized, data-driven approach that uses technology and advanced analytics to transform point-of-care practice. In conclusion, this Research Topic, “*The Impact of Pharmacokinetics and Pharmacodynamics on Antimicrobial Stewardship*,” strongly concludes that PK/PD science is not merely a reactive tool in the fight against AMR but also forms the basis for proactive, predictive, and personalized therapy.
